# One-against-All Weighted Dynamic Time Warping for Language-Independent and Speaker-Dependent Speech Recognition in Adverse Conditions

**DOI:** 10.1371/journal.pone.0085458

**Published:** 2014-02-10

**Authors:** Xianglilan Zhang, Jiping Sun, Zhigang Luo

**Affiliations:** 1 Science and Technology on Parallel and Distributed Processing Laboratory, School of Computer Science, National University of Defense Technology, Changsha, Hunan, China; 2 State Key Laboratory of Pathogen and Biosecurity, Beijing Institute of Microbiology and Epidemiology, Fengtai District, Beijing, China; 3 David R. Cheriton School of Computer Science, University of Waterloo, Waterloo, Ontario, Canada; 4 Voice Enabling Systems Technology, Waterloo, Ontario, Canada; King Saud University, Saudi Arabia

## Abstract

Considering personal privacy and difficulty of obtaining training material for many seldom used English words and (often non-English) names, language-independent (LI) with lightweight speaker-dependent (SD) automatic speech recognition (ASR) is a promising option to solve the problem. The dynamic time warping (DTW) algorithm is the state-of-the-art algorithm for small foot-print SD ASR applications with limited storage space and small vocabulary, such as voice dialing on mobile devices, menu-driven recognition, and voice control on vehicles and robotics. Even though we have successfully developed two fast and accurate DTW variations for clean speech data, speech recognition for adverse conditions is still a big challenge. In order to improve recognition accuracy in noisy environment and bad recording conditions such as too high or low volume, we introduce a novel one-against-all weighted DTW (OAWDTW). This method defines a one-against-all index (OAI) for each time frame of training data and applies the OAIs to the core DTW process. Given two speech signals, OAWDTW tunes their final alignment score by using OAI in the DTW process. Our method achieves better accuracies than DTW and merge-weighted DTW (MWDTW), as 6.97% relative reduction of error rate (RRER) compared with DTW and 15.91% RRER compared with MWDTW are observed in our extensive experiments on one representative SD dataset of four speakers' recordings. To the best of our knowledge, OAWDTW approach is the first weighted DTW specially designed for speech data in adverse conditions.

## Introduction

This paper studies language-independent (LI) with light weight speaker-dependent (SD) automatic speech recognition (ASR) in adverse conditions, such as noisy environment and bad recording condition of too high or low volume. As speech is the primary method for human communication [1], the fast development of communication devices has attracted much enthusiasm for research in ASR over the past decades [2]. ASR recognizes human speech using computer algorithms without the involvement of humans [3]. It is essentially a pattern recognition process. Taking one pattern, i.e. the speech signal, ASR classifies it as a sequence of previously learned patterns [4].

LI means that a speech recognition algorithm can recognize speeches in kinds of different languages. LI SD ASR has wide applications. Voice dialing on mobile communication devices, menu-driven recognition, and voice control on vehicles and robotics should be treated as LI SD ASR applications. This is because: 1. these applications have widely usage so that they should be language independent (LI) rather than be limited to specific language(s); 2. because these applications should be used not only online but also off-line, they can be developed as speaker-dependent (SD) applications. Many corporations, such as Google and Microsoft, have developed mature speaker-independent (SI) ASR applications. However, most of the current applications are all language-dependent (LD). Such LD SI ASRs are based on Hidden Markov Model (HMM) [5], the accuracy of which relies on the amount of training data. That is, the more the training data are available to train a phone or word model, the more accurate the recognition will be. However, due to excessive time, storage, and cost factors associated with the collection of multi-language training data, lack of sufficient training data in non-English language means that those mature SI ASR applications cannot achieve good accuracy when used for non-English ASR. Furthermore, all of the contact information in personal mobile devices has to be uploaded to remote ASR servers when performing SI ASR, which may cause an inherent risk of loss of personal information. For safety reasons, it is better to store such information on one's own device rather than to upload it to remote servers. Considering difficulty of obtaining training data for seldom used English words and (often non-English) names and personal privacy, LI with light-weighted SD ASR is a promising option to solve the problem.

Statistical model based and template based technologies are two main ASR categories. Hidden Markov model (HMM) is the most popular statistical model based approach. Baum and his colleagues developed the mathematics behind the HMM in the late 1960s and early 1970s [6]. Then, HMM was firstly applied to speech recognition by Baker at CMU, and by Jelinek and his colleagues at IBM in 1970s [6]. Since the mid-1980s, HMM has become widely implemented in speech processing applications [1]. Generally speaking, after HMM training and recognition process, one speech signal tested can be correlated to a certain text. HMM is more flexible in large vocabulary systems, and achieves better performance in SI cases [7]. On the other hand, Dynamic Time Warping (DTW) is the most well-known speech recognition technique among the template based technologies. In 1968, Vintsyuk proposed the use of dynamic programming (DP) methods for the element-by-element recognition of words, and such DP methods performs perfectly in an experimental test of SD case [8]. Thereafter, this method has been incorporated in many speech processing applications [9]–[16]. DTW uses a DP alignment process to find the similarity between two speech signals, which excellently measures the similarity between speech signals in SD cases [17].

Because storage space of mobile devices and personal information are limited, our goal is to develop LI with light-weighted SD ASR algorithm. In such algorithm, we use **only one sample** for each word as training data. Considering that HMM needs sophisticated implementation of large-scale software and lots of training data [18], and dynamic time warping (DTW) aims at small-scale embedded systems with its simplicity in hardware implementation [3], we choose DTW in this work.

There are many DTW variations. Some are designed for fast computing, and the others are designed for improving their performances. Many variations have been proposed for accelerating DTW computing process [19]. Be it lower bounding measure [20], global constraint region usage [21], multi-scale DTW [22], or any other combination of the first two methods [23], they are all based on constraint algorithms in an iterative fashion [24]. That is, speeding up DTW process at the expense of accuracy. On the other hand, considering that DTW gives each time frame an equal weight to align two time series, the authors of [25] and [26] introduced two weighted DTW methods to avoid potential misclassification caused by equal weight. These two methods weight nearer neighbors differently depending on the phase similarity between a training time frame and a testing time frame. It is noted that these weighted DTW do not decrease time complexities. We have developed confidence index dynamic time warping (CIDTW) [27] and merge-weighted dynamic time warping (MWDTW) [28] methods of fast and accurate speech recognition for clean speech data. Both methods involve a merging step that merges adjacent similar time frames in one speech signal and then performs DTW on merged speech data. The merging step can significantly improve the running time of the speech recognition process. Using our CIDTW and MWDTW, we speed up DTW recognition process with improved accuracy. However, CIDTW and MWDTW do not work well on noisy and badly recorded speech data. Here, badly recorded data means that the speaker's volume is too high or low.

Through experiment, we have found that the merging step in our CIDTW and MWDTW is the main reason why these two methods are not able to work on noisy and badly recorded data. Specifically, merging adjacent similar time frames requires determining a time frame merging threshold. If some speeches contain noise or miss the information of top waves when recording in a very high volume, this merging threshold will probably not show the real merging baseline. As a result, the merging step may lead to wrong classifications. Therefore, novel methods are needed to address the challenge of accurate and fast speech recognition in noisy and bad recording conditions.

In order not to lose recognition accuracy for noisy and badly recorded speech data, we develop a novel one-against-all weighted dynamic time warping (OAWDTW) algorithm. Unlike our former CIDTW and MWDTW weighting scheme, OAWDTW defines one-against-all index (OAI) for each time frame of training data, then applied OAIs into general DTW process to tune the final alignment score and to find the similarity between merged training and testing data. We build a representative dataset recorded by 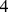 speakers under different recording conditions. Compared with original DTW, our OAWDTW achieves better accuracy both on clean data with 0.5% relative reduction of error rate (RRER) and on noisy data with 7.5% RRER.

To the best of our knowledge, our method is the first weighted DTW specially designed for noisy and not well recorded speech data.

## Materials and Methods

### 1 Dynamic Time Warping Algorithm

There are variations of voice and speed for a single word even if such word is spoken by the same person many times. Dynamic time warping (DTW) can detect such variations.

Suppose that two input speech signals, 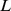 with length 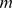 and 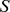 with length 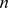, vary in time. Since our method is built upon it, we illustrate here the complete DTW algorithm which contains two processes, DTW matrix calculation and optional DTW alignment path search. The value of elements in DTW matrix is acquired by using the formula at the tenth step of Algorithm 1 in Table 1, the last element of the whole matrix represents the similarity between 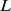 and . The smaller the value of this last element is, the closer 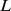 and 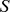 would be. After filling the whole matrix, the alignment path will be acquired through backtracking of the DTW matrix from the last element. Usually, we only need to know the final alignment score of DTW. In our paper, we need to use steps 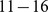 of Algorithm 1 in Table 1 to acquire the alignment details between two speech signals.

**Table 1:** Algorithm 1: Dynamic Time Warping.

**Require:** speech sequences 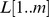 , 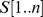 .	
1: 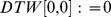	
2: **for** i : = 1 to 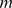 **do**	12: **if** 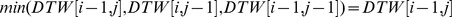 **then**
3: 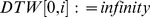	13: 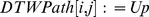
4: **end for**	14: **else if** 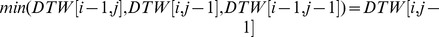 **then**
5: **for** i : = 1 to 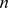 **do**	15: 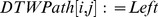
6: 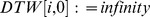	16: **else**
7: **end for**	17: 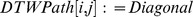
8: **for** j : = 1 to 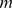 **do**	18: **end if**
9: **for** j : = 1 to 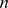 **do**	19: **end for**
10: 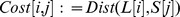 // 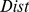 represents the Euclidean distance	20: **end for**
11: 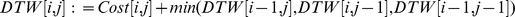	21: **return** 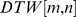

### 2 One-Against-All Weighted Dynamic Time Warping

The novel one-against-all weighted dynamic time warping (OAWDTW) can process spectrograms or mel frequency cepstral coefficient (MFCC) acoustic features of audio files. Spectrogram and MFCC are both visually representation of acoustic speech signal. In this paper, we use MFCC as input. To make the description of the OAWDTW more clearly, we will specify the input speech file format as MFCC in the rest of the paper. Essentially, MFCC can be treated as a matrix. The MFCC of one speech signal is actually multi-dimensional feature vectors, which show the change of periodic signal's frequency, amplitude, etc.

Using OAWDTW to perform speech recognition, we only need to **record** each word for **one time** as training data. For clarity, let us first define several terminologies to describe a MFCC and its time frames:

1. **training MFCC**: MFCC of training speech signal.
2. **testing MFCC**: MFCC of testing speech signal.
3. **time frame**: a multi-dimensional feature vector in training or testing MFCC, which represents the feature distribution over a certain time period.

**Figure 1:**
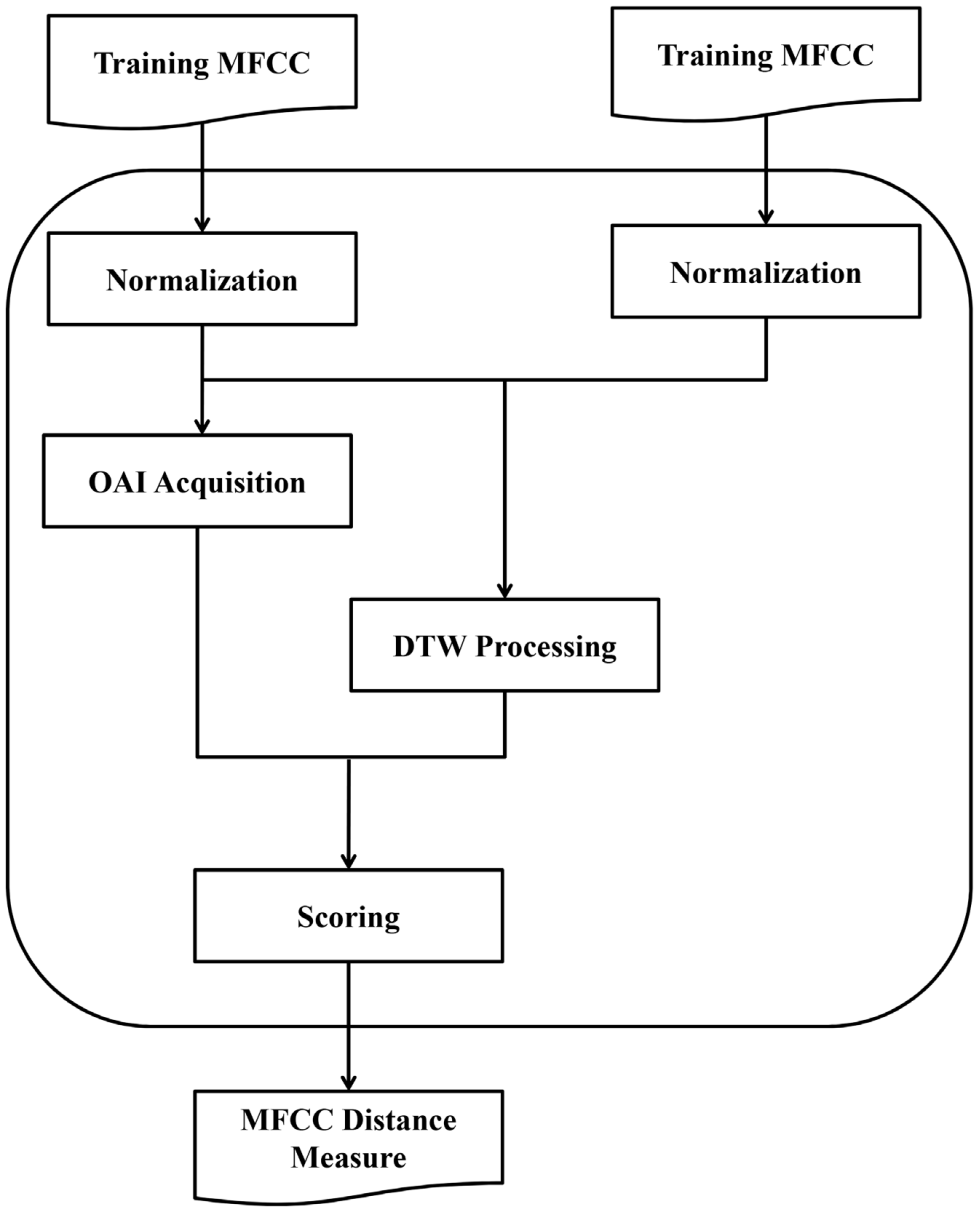
Summary of the proposed one-against-all weighted dynamic time warping (OAWDTW) approach. First, OAWDTW normalizes training MFCCs and testing MFCCs. Then, OAWDTW acquires the one-against-all index (OAI) of each time frame in training MFCCs. Third, OAWDTW performs dynamic time warping alignment between a normalized training MFCC and a normalized testing MFCC. Forth, OAWDTW applies OAIs of aligned time frames in normalized training MFCC to tune the final score.

As illustrated in Figure 1, our OAWDTW contains four steps:

1. Normalize training and testing MFCC.
2. Acquire the one-against-all index (OAI) of each training MFCC by using DTW.
3. Find aligned path of testing MFCC and training MFCC by using DTW.
4. Score the similarity between testing and training MFCC by applying the OAIs into the alignment path acquired in step 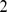.

The dynamic time warping (DTW) algorithm is the core of the OAWDTW. Generally speaking, OAWDTW finds the aligned path of training and testing MFCC by using DTW (step 3), and then applies OAI as weights of aligned time frames to adjust the final aligned score (step 4). Step 1 and 2 preprocess original MFCCs for next steps. Each step of OAWDTW is described in greater detail in the following subsections.

#### 2.1 MFCC Normalization

**Figure 2:**
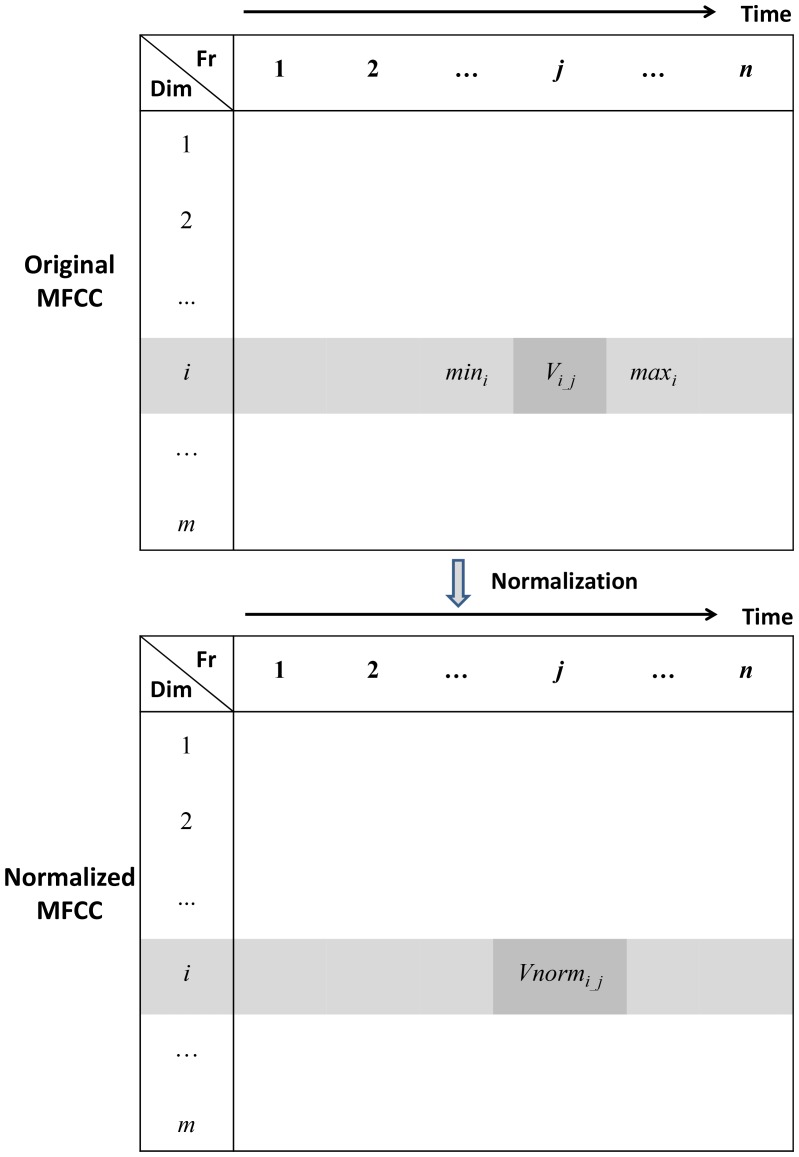
The illustration of MFCC normalization. In table header, ‘Fr’ represents time frame, and ‘Dim’ means dimension. A MFCC is represented by a 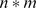 matrix. This matrix is constituted by 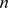 time frames where each time frame is represented by a 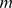 dimensional vector. Therefore, each dimension has 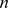 values, which will be normalized into the range between −1 and 1 after the MFCC normalization step.

MFCC is a high-dimensional vector. The values in each dimension have different ranges and scales. In order to make comparison meaningful, these values in same dimension need to be normalized between 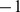 and 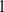. Here, we adopt the normalization process proposed by authors of [30]. Here we suppose that 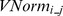 is the 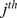 normalized value in the 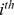 dimension, 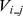 is the 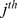 value in the 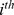 dimension, 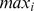 is the maximum value of the 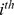 dimension, 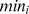 is the minimum value of the 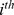 dimension. As shown in Figure 2, a MFCC is represented by a 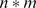 matrix, which contains 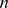 frames where each frame is represented by a 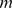 dimensional vector. A normalized MFCC is acquired after using equation 1:
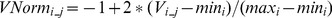
(1)


#### 2.2 One-Against-All Index of training MFCC

**Figure 3:**
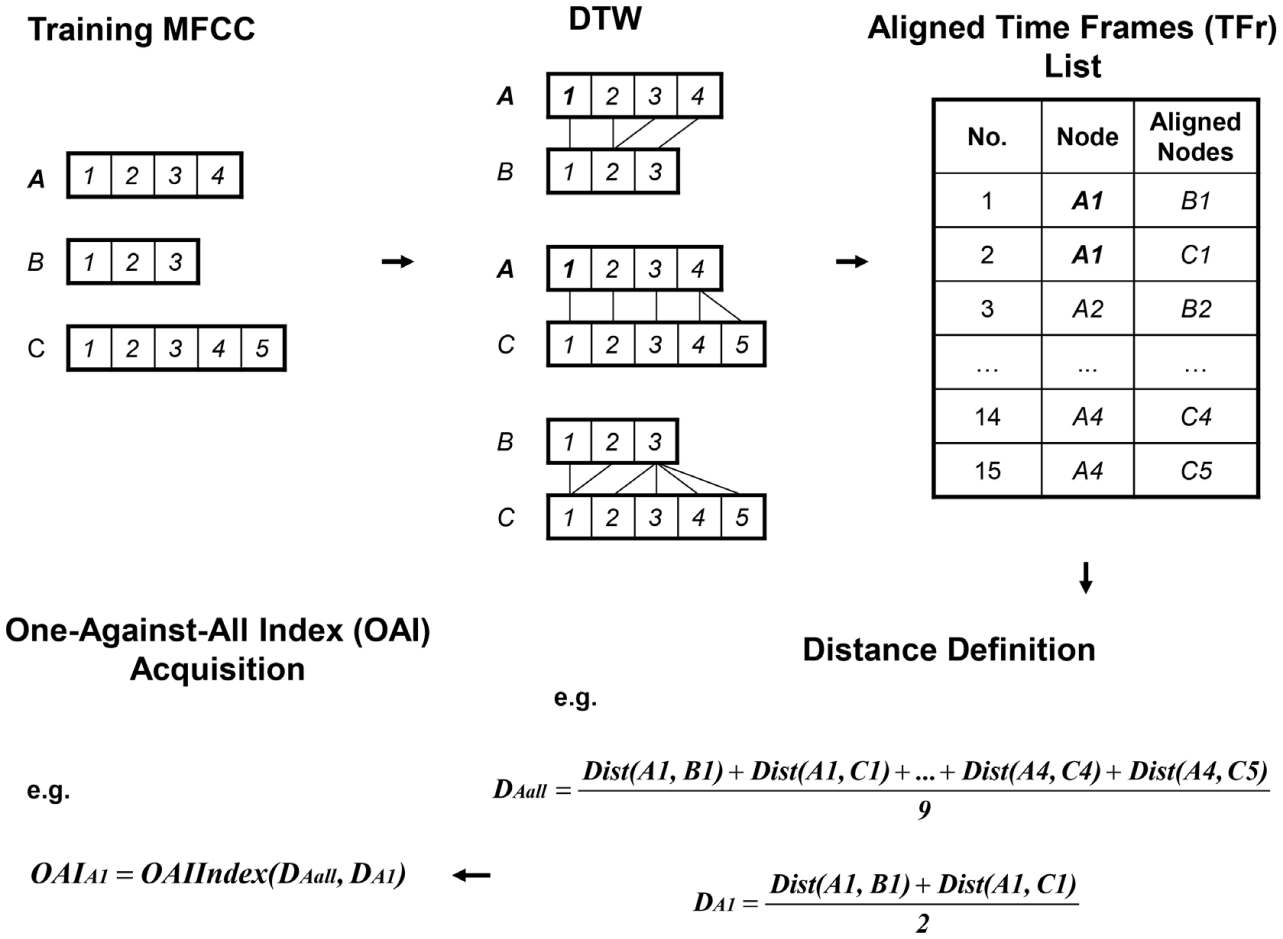
The illustration of one-against-all index (OAI) acquisition. Suppose that there are three training MFCCs. We take the node 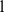 of training MFCC 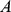 (bold and italic letter) as example. The 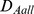 is acquired by using all of the distances among nodes in 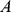 and their aligned nodes. The 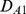 is acquired by using all the distance between node 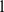 in 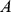 and its aligned nodes.

An illustration of one-against-all index (OAI) acquisition process is shown in Figure 3.

Specifically, in a training MFCC data set, we first do general DTW between every pair of training MFCCs so that we acquire all aligned pairs of time frames. Then we calculate the average distance among time frames in one training MFCC and their aligned time frames. Let us denote this distance by 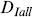, where 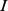 represents the 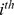 training MFCC. 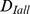 is the ‘All’ in our method's name ‘One-Against-All’. For each time frame 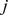 of this training MFCC, we also calculate the average distance between it and its aligned time frames, which is denoted by 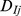, which is the ‘One’ in method's name ‘One-Against-All’.

Now the OAI of time frame 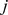 in the 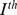 training MFCC is defined as function 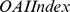:
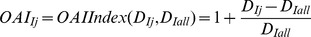
(2)


In this way, for a time frame 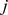 in the 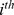 training MFCC, if its 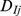 equals the average distance among time frames in the 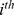 training MFCC, its OAI will be 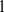. If its 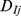 is larger than average, its OAI is slightly larger than 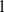. If its 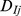 is less than average, its OAI is slightly less than 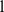.

The idea behind the above definition is that we take the global average of time frame to time frame alignment distances as the basis of measurement. If one time frame's average distance from its aligned time frames is shorter than the global average of the time frames in one specific training MFCC, that means this time frame is quite similar to time frames in the same training MFCC. That gives lower confidence to it as model element. Therefore, its confidence value is smaller than 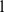. On the other hand, if a time frame's average distance from its aligned time frames is longer than the global average, that means this time frame is quite different from time frames in a same training MFCC. That gives higher confidence to it as model element. Hence its confidence value is larger than 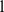.

#### 2.3 Dynamic Time Warping Aligned Path of Normalized Training and Testing MFCC

By backtracking the array 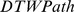 in Algorithm 1 in Table 1 of one normalized training MFCC and one normalized testing MFCC, we receive their time frames' alignment path. Suppose that the time frame lengths of the normalized training and testing MFCC is 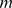 and 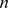, respectively, where 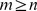. Then we use 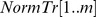 to store the normalized training MFCC time frames, and 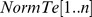 to store the normalized testing MFCC time frames. Since 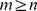, the length of the two MFCC's aligned path is 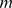, where a time frame in the training MFCC may be aligned with two to more time frames of the testing MFCC. The aligned time frame orders of 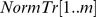 and 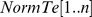 are stored in 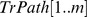 and 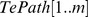, respectively. These arrays will be used in the next MFCC similarity scoring process.

#### 2.4 Score the Similarity between Normalized Training and Testing MFCC

The similarity scoring process of the proposed OAWDTW is shown in Algorithm 2 in Table 2. Here, as the same as Algorithm 1 in Table 1, the 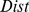 function is the Euclidean distance between a time frame in training MFCC and a time frame in testing MFCC. We use the 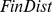 to represent the similarity between one normalized training and testing MFCC. The smaller the 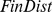 is, the more similar the training MFCC and testing MFCC are. After using the OAI of each time frame in one specific training MFCC to readjust the DTW score, we improve the alignment accuracy.

**Table 2:** Algorithm 2: Similarity Score of Normalized Training and Testing MFCC.

**Require:** the *i^th^* training MFCC *I*, aligned path	3: *Score*[*k*]: = OAI_Ik_*Dist(*TrPath*[*k*],*Tepath*[*k*]
*TrPath*[1..*m*] and *TePath*[1.*m*]	4: *FinDist*: = *FinDist*+*Score*[*k*]
1: *FinDist*: = 0	5: **end for**
2: **for** *k* : = 1 to *m* **do**	6: **return** *FinDist*

## Results

### Data Preparation

Generally speaking, our data preparation includes 4 steps:

1. Audio files record;
2. MFCC generation;
3. MFCC end point detection;
4. MFCC dimension extraction.

More details are described as follows.

**Figure 4:**
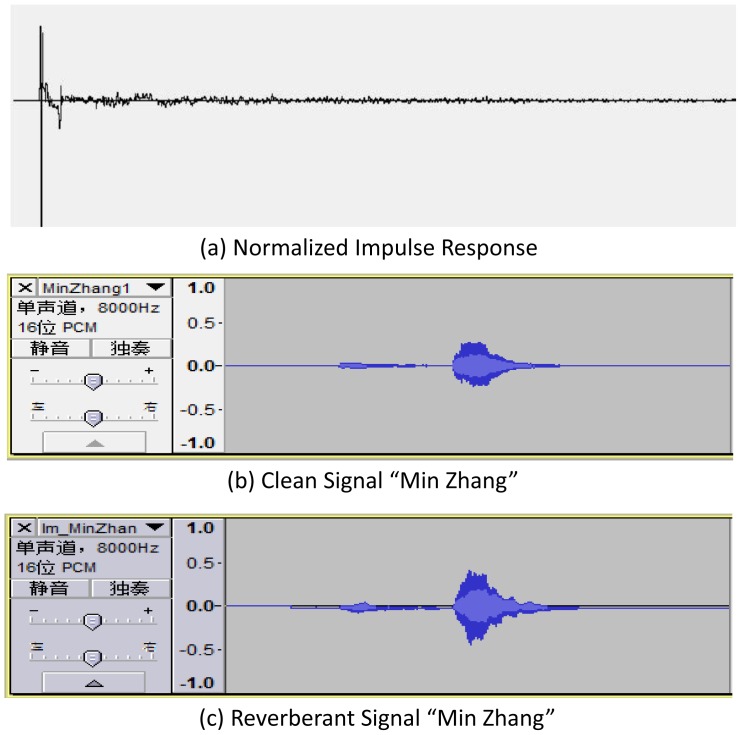
The samples of clean and reverberant signals of audio “MinZhang”.

To test whether OAWDTW is suitable for language independent (LI) speaker dependent (SD) automatic speech recognition (ASR), we need to have a multi-language speech corpus in which each word is recorded for at least two times – one as training data, the other as test data. However, most of the current public speech corpora are built for SI ASR. That is, these corpora only contain sentences (a few words) in one or two languages while each sentence/word is recorded once. Fortunately, as we know that name is made up of words, we choose the most representative names in their respective countries. Thus, these different names can be treated as the representation of commonly used words in multiple languages. Three females and one male use the Audacity software to manually record a total number of 65 different names and terms of address in English, Chinese, German, French, Arabic, and Korean. Each speaker records in different environment and recording situation and repeats each name or term of address 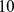 times. As shown in Table 3, two speakers record in a quiet environment (speaker 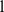 and 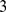). To test the robustness of OAWDTW against noise corrupted data, we use the Mardy reverberant noise database [31] to add reverberant noise to the clean speech recorded by speaker 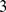. The Mardy database was developed to test denoising algorithms. Since we currently do not apply denoising filtering, we do not need the information provided by the Mardy database with respect to the distances between source and microphones, the microphone array channels and loud speaker positions. Therefore, we randomly select one impulse response to simulate noise corruption through convolution as described in [32]. The process is as follows: we normalize the impulse response to have maximum value of 1, and convolve the speech data with the normalized impulse response. Figure 4(a), 4(b) and 4(c) show the normalized impulse response, the original clean speech and corrupted reverberant speech. Speaker 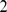 records in a noisy environment with a consistent background sound, and speaker 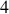 uses a very high volume which often gets over the largest value of short integer (about 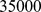 in C language when programming to deal with the audio files) so the top of the waves are clipped. The recording settings are 8 kHz, mono channel, 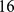 bits PCM. The name list is shown in Table 4. We first record some Chinese names. In order to test whether our method is compatible with multiple languages, we introduce some French, German, Arabic, Korean, English, along with English-Chinese names (first name is English, while last name is Chinese). Considering that our goal is to enhance name recognition accuracy, especially for Chinese words, we introduce 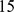 different Chinese terms to address ‘father’, ‘mother’, ‘son’, ‘daughter’, ‘grandparents’, etc. Please email zhangxianglilan@gmail.com for these raw data. These Chinese terms are represented by PinYin, and their meanings are listed in paired parentheses.

**Table 3:** Recording Condition Description.

No.	Speaker1	Speaker2	Speaker3	Speaker4
Recording Environment	Quiet	Noisy	Reverberant	Quiet
Recording Volume	Normal	Low	Normal	Too Loud

**Table 4:** List of Recorded Names.

No.	1	2	3	4	5
Name	Celine Dion	Elizabeth Taylor	Jim Carrey	Julia Roberts	Austin Zhao
No.	6	7	8	9	10
Name	Tom Hu	Nancy Zhang	Angela Chan	Annie Tao	Ying Lee
No.	11	12	13	14	15
Name	Chunlei Zhao	Cong Jiang	Di Tang	Ruijie Shen	Wendan Tao
No.	16	17	18	19	20
Name	Wenqiang Tian	Xiaojin Gu	Xuefeng Hu	Yang Wang	Yin She
No.	21	22	23	24	25
Name	Yu Jiang	Xing Wang	Xianglilan Zhang	Yanmei Li	Changqin Chu
No.	26	27	28	29	30
Name	Min Zhang	Chaobi Li	Chen Zhang	Lin Zhang	Luyang Zhang
No.	31	32	33	34	35
Name	Muhammad Yunus	Omar Al-Khayyam	Fatema Mernissi	Zakaria Botros	Girias Bangalore
No.	36	37	38	39	40
Name	André Rieu	Edouard Manet	Cécile Chaminade	Aimée Allen	Grégoire Boissenot
No.	41	42	43	44	45
Name	Achim Steiner	Albrecht Dürer	Kai Tak	Nicholas D. Kristof	Marcel Wanders
No.	46	47	48	49	50
Name	An Cho-yeong	Ch'a Min-su	Heo Chang-heui	Im Seon-keun	Kim-Chu-ho
No.	51	52	53	54	55
Address	Ba Ba	Die	Er Zi	Fu Ren	Lao Gong
(Meaning)	(Papa)	(Father)	(Son)	(Wife)	(Husband)
No.	56	57	58	59	60
Address	Lao Po	Ma Ma	Nai Nai	Niang	Nv Er
(Meaning)	(Wife)	(Mama)	(Grandma)	(Mother)	(Daughter)
No.	61	62	63	64	65
Address	Tai Tai	Xiao Wang	Xiao Zhang	Ye Ye	Zhang Fu
(Meaning)	(Wife)	(Mr./Ms. Wang)	(Mr./Ms. Zhang)	(Grandpa)	(Husband)

Referring to Chapter 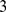 of HTK manual [29], The HCopy function in HTK converts the ‘.wav’ audio files into ‘.mfc’ files. When using HTK, the frame period is 25 msec, fast Fourier transform (FFT) uses a Hamming window. A coefficient of 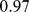 first order pre-emphasis is applied to a signal. Filterbank has 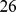 channels and outputs 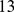 MFCC coefficients. At last, we use HList function in HTK to convert binary.mfc files into text formats, so that we can treat converted files as inputs of our OAWDTW method.

For the above generated MFCCs, we write an end point detection program, which removes silence at the beginning and end of recording, and long silence in the middle of ‘.wav’ file. Please email zhangxianglilan@gmail.com for this program. Given one 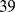 dimensional MFCC, its first 13 dimensions are the MFCC parameters, its next 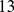 dimensions are deltas derived from the MFCC, and its last 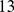 dimensions are the double deltas (accelerations). Considering that the last 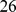 dimensions are for training purpose in HMM rather than for time series DTW alignment, our method uses the first 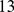 dimensions to represent the MFCC feature vector of one audio file.

### OAWDTW VS. DTW and MWDTW

Consider that our MWDTW is developed from CIDTW, we use the original DTW, MWDTW and our OAWDTW to test the 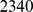 recordings of 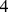 speakers. For each speaker, the recordings include 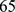 names with each name repeated 10 times. According to our former experiments on clean recording data [28], HMM is worse than the original DTW in terms of performance. Thus, it is unnecessary to compare our OAWDTW with HMM here. To make our experiments more convincing, cross validation approach is applied in this paper. Given ten times of recordings for each name, one audio file of a certain name is randomly picked out as training data, the other nine files are testing data. Therefore, ten times of cross validation experiments have been done for each dataset, and each cross validation experiment has its unique training data.

To demonstrate the importance of MFCC normalization for time series DTW, we tested the original DTW by using 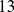 dimensional MFCC with normalization, 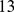 dimensional MFCC without normalization. The results are shown in Table 5. The overall average accuracy of four speakers in the last column are highlighted in a italic font. The original DTW achieves a better result by using normalized 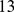 dimensional MFCC as input. Since speaker 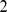 records her audio files in a noisy environment, the quality of her speech is worse than the quality of the speeches given by the other three speakers. Under reverberant environment, the quality of the speech of speaker 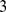 is worse than the quality of the speeches of speaker 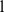. Because speaker 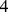 records his audio files using a too high volume, the quality of his speech is worse than the quality of the speeches given by speaker 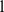. Generally speaking, compared with volume and reverberant environment, noise has much more impact on speech recognition accuracy.

**Table 5:** Accuracy (%) by using original DTW on different (non)normalized dimensional MFCC.

	Speaker1	Speaker2	Speaker3	Speaker4	*Overall*
13 Norm	98.1006	81.1965	94.1880	94.3589	*91.9610*
13 NonNorm	98.1006	72.6496	93.8461	91.2820	*88.9696*

In the first column, numbers represent dimensions, ‘Norm’ means normalized MFCC, and ‘NonNorm’ means unnormalized MFCC.

Considering that original DTW achieves a better accuracy by using normalized 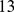 dimensional MFCC, we use such MFCC as the input of our MWDTW and OAWDTW. We define a performance measure to evaluate the effectiveness of our OWADTW before comparing it with original DTW. This performance measure is called relative reduction of error rate (RRER), of which the definition is described in Eq.3 :
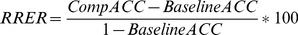
(3)Here, ‘CompACC’ means the accuracy of a compared method that is OAWDTW in our paper. ‘BaselineACC’ means accuracy of an established method that is the original DTW in our paper.

As shown in Table 6, the accuracies of MWDTW are worse than OAWDTW. Specifically, the average accuracy of MWDTW is 6.2% worse than DTW. As already mentioned in the section of introduction, the merging step in MWDTW is thought to be the reason that MWDTW cannot achieve a good accuracy when doing speech recognition under noisy and bad recording conditions. Most importantly, OAWDATW achieves better accuracy than DTW. Especially, under quiet environment and good recording condition (Speaker 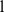), OAWDTW improves the accuracy by about 0.18% compared with the original DTW and acquires 10% RRER on average. Under noisy environment and low volume of speech recording condition (Speaker 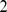), OAWDTW improves recognition accuracy by about 1% compared with the original DTW and acquires a 5.45% RRER. Under reverberant environment and normal recording condition (Speaker 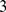), OAWDTW improves the accuracy by about 0.52% and acquires a 8.82% RRER. Under quiet environment and bad recording condition (Speaker 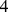), OAWDTW improves the accuracy by about 0.5% and acquires a 9.09% RRER. As DTW already does almost perfect speech recognition under quiet environment [27] [33]–[34], it is likely that we will not get any improvement by using OAWDTW. Thus it is encouraging that our OAWDTW achieves a little better recognition accuracy for bad recording condition. For average accuracy, OAWDTW achieves 0.56% better accuracy than original DTW and acquires a 6.97% RRER. Compared with DTW, OAWDTW accomplishes better speech recognition, especially under noisy environment. It means that OAWDTW is more robust and more accurate than DTW.

**Table 6:** DTW accuracy (DTW Acc) and OAWDTW accuracy (OAWDTWAcc)(%) comparison, and OAWDTW relative reduction of error rate (OAWDTW RRER) (%) based on DTW.

	Speaker1	Speaker2	Speaker3	Speaker4	*Overall*
DTW Acc	98.1006	81.1965	94.1880	94.3589	*91.9610*
MWDTW Acc	98.1799	65.8120	96.7521	81.1965	*85.4851*
OAWDTW Acc	98.2906	82.2222	94.7009	94.8718	*92.5214*
OAWDTW RRER (compared with DTW)	10.0032	5.4548	8.8248	9.0922	*6.9710*
OAWDTW RRER (compared with MWDTW)	6.0821	47.9999	−63.1547	72.7274	*15.9137*

## Discussion

In this paper, we introduce a novel one-against-all weighted dynamic time warping (OAWDTW) to provide efficient automatic speech recognition service in noisy environment and bad recording conditions where the volume is too high or too low. By testing one representative dataset of four speakers' all 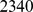 recordings in different environments and recording conditions, OAWDTW gives improved results compared with DTW and MWDTW, especially under noisy environment. Our OAWDTW is the first weighted DTW variation specially designed for speech data in different recording environment and conditions.

Our goal is to develop simpler and more efficient methods. We are in the process of improving the speed of our algorithm and making it applicable as an efficient robust light weight SD ASR service for real-time language independent applications with small vocabulary and limited storage space, such as voice dialing on mobile devices, menu-driven recognition, and voice control on vehicles and robotics, especially under noisy environment and bad recording conditions. In addition, we focus on using this method to analyze spectrogram rather than MFCC, and hopefully achieve a comparable result by using pure spectrogram.
